# Soil Disturbance of Plateau Zokor (*Eospalax baileyi*) Promotes the Stability of Alpine Plant Communities

**DOI:** 10.3390/plants14182830

**Published:** 2025-09-10

**Authors:** Xidong Zhu, Feiyang Xue, Zhiying Zhang, Rui Dong, Limin Hua, Guohui Ye

**Affiliations:** 1 Key Laboratory of Grassland Ecosystem of the Ministry of Education, Engineering and Technology Research Center for Alpine Rodent Pest Control of National Forestry and Grassland Administration, College of Grassland Science, Gansu Agricultural University, Lanzhou 730070, China; zhuxidong0510@163.com (X.Z.); 18738373198@163.com (F.X.); zhangzy@st.gsau.edu.cn (Z.Z.); dongrui_gsau@163.com (R.D.); 2 Key Laboratory of Grassland Rodent Ecology and Rodent Pest Control at Universities of Inner Mongolia Autonomous Region, Key Laboratory of Grassland Resources, Ministry of Education, College of Grassland, Resources and Environment, Inner Mongolia Agricultural University, Hohhot 010018, China

**Keywords:** plant community stability, spatial disturbance gradient, plateau zokor, compositional convergence, indicator species analysis, soil–vegetation interactions, alpine meadow ecosystem

## Abstract

Alpine meadows on the Tibetan Plateau experience chronic, fine-scale disturbances from the plateau zokor (*Eospalax baileyi*), a subterranean rodent that alters soil and vegetation structure through persistent burrowing and mounding. While classical theory predicts that plant community stability peaks at intermediate disturbance levels, this may not apply under spatially heterogeneous disturbance regimes. We assessed community stability across a five-level zokor disturbance gradient using a multi-indicator framework integrating compositional variability (average variation degree, AVD), co-occurrence-based cohesion, indicator species analysis, and boosted regression tree (BRT) modeling. Stability (1−AVD) peaked under extreme disturbance, alongside reduced indicator species richness and the dominance of disturbance-tolerant taxa. Increased cohesion suggested stronger species associations. Drivers of stability shifted from plant attributes under low disturbance to soil constraints (bulk density and moisture) under high disturbance. These results challenge the intermediate disturbance–stability paradigm and suggest that abiotic filtering can promote compositional convergence and structural stability. Our findings highlight the importance of spatial disturbance patterns in shaping community resilience and provide early warning indicators and targeted guidance for managing alpine grasslands under subterranean disturbance.

## 1. Introduction

The alpine meadows of the Tibetan Plateau, covering nearly one-quarter of its total area, represent one of the most extensive and ecologically complex ecosystems on Earth [1,2,3]. These grasslands not only harbor rich biodiversity but also provide essential ecosystem services, including carbon storage and water regulation for millions of downstream inhabitants [1,3,4,5]. At the core of their ecological resilience lies the stability of plant communities—the capacity to maintain structural and functional integrity in the face of environmental fluctuations and disturbances [6,7,8]. In an era of accelerating global change—including climate warming, altered precipitation regimes, land-use intensification, and nutrient deposition—identifying the ecological mechanisms that underpin this stability is of urgent importance [6,7,8,9].

Among the key natural engineers of this landscape is the plateau zokor (*Eospalax baileyi*), a subterranean rodent whose persistent burrowing and mounding activities dramatically alter the physical and chemical properties of soil [10,11]. These behaviors create marked spatial heterogeneity, generating a mosaic of disturbance intensities across the landscape [12,13]. The resulting disturbance gradient—reflected particularly in the inter-mound vegetation matrix—introduces variability in community composition and ecological processes across spatial scales, making it an ideal system for studying community stability under patchy disturbance regimes [7,12,13,14].

The classical Intermediate Disturbance Hypothesis (IDH) posits that community stability peaks at intermediate levels of disturbance [15]. However, the spatially complex, multi-intensity disturbance regime driven by plateau zokor activity challenges this paradigm [11,16]. This discrepancy may arise because the IDH assumes that disturbances are spatially homogeneous and occur at scales that allow for a balance between competitive exclusion and colonization, thereby producing a unimodal stability–disturbance relationship. In contrast, zokor disturbance is patchy, recurrent, and highly heterogeneous in space, creating a fine-scale mosaic of microhabitats with different recovery trajectories. Such spatial heterogeneity can maintain or even enhance stability under high disturbance by allowing disturbance-tolerant species to persist in severely affected patches, while undisturbed patches act as refugia for other taxa. Field observations in zokor-affected grasslands often reveal unexpectedly high levels of community robustness, even under strong disturbance, suggesting that mechanisms beyond the IDH—such as rapid colonization by disturbance-tolerant species, restructured interaction networks, or adaptive shifts in resource-use strategies—may play key stabilizing roles [17,18,19].

This study aims to address three interrelated questions: (1) How do compositional stability and interaction network robustness vary along the continuous gradient of zokor disturbance? Do these patterns support or challenge the predictions of the IDH? (2) Which species serve as reliable indicators of different disturbance intensities? How do their distributions reflect shifts in community structure? (3) What are the dominant biotic and abiotic drivers of stability at different disturbance stages? Do these drivers shift across the gradient in ways that reveal thresholds or stabilizing feedbacks?

Addressing these questions, however, faces fundamental methodological challenges inherent in quantifying stability under spatially heterogeneous disturbance regimes. Specifically, our understanding of how plant community stability responds to such spatially stratified disturbance gradients, particularly within inter-mound grasslands, remains limited due to several fundamental challenges [20,21,22]. First, ecological stability is a multidimensional concept encompassing the constancy of species composition, the robustness of species interaction networks, and the persistence of ecosystem functioning [23,24]. Most studies rely on single metrics (e.g., species richness or biomass), which fail to capture compensatory dynamics such as functional redundancy or shifts in species dominance, thereby obscuring a holistic understanding of community resilience [23,25]. Second, species do not play static roles across disturbance gradients. In lightly disturbed grasslands, dominant grasses may underpin stability, whereas under more intense disturbance, species with high tolerance or adaptive traits may become critical to community persistence [26,27]. Capturing these dynamic shifts in species’ functional importance is essential for understanding the mechanisms of stability [25,28]. Third, the relative importance of biotic versus abiotic drivers is expected to vary along the disturbance continuum. Biological processes (e.g., competition and diversity) and physical constraints (e.g., soil compaction and moisture limitation) interact in nonlinear ways, posing substantial challenges to identifying causal relationships and shifts in dominant stabilizing forces [29,30].

To address the multifaceted challenges of quantifying plant community stability under spatially heterogeneous zokor disturbance, this study employs a comprehensive, multi-indicator analytical framework designed to capture stability from multiple ecological dimensions [31,32]. Compositional stability is assessed using the average variation degree (AVD), a sensitive metric that quantifies fluctuations in species abundances across spatial and temporal scales, thereby reflecting the constancy of community composition beyond traditional diversity indices [33,34]. This approach enables the detection of subtle compensatory dynamics, such as shifts in species dominance and functional redundancy, which are critical for understanding resilience [35]. The structural robustness of species interactions within the community is evaluated through community cohesion indices derived from species co-occurrence networks. These indices quantify the strength and persistence of species associations, offering insights into how ecological networks reorganize and maintain function amid disturbance [36,37]. Such network-level perspectives are essential for elucidating mechanisms of stability that transcend simple compositional metrics [38]. Recognizing that species’ sensitivity and roles vary along disturbance gradients, we apply specificity–occupancy (SPEC-OCCU) analysis to identify indicator species characteristic of distinct disturbance intensities [39,40]. This method combines species habitat specificity and occupancy frequency to reveal shifts in community structure and identify taxa pivotal to stability at different stages of disturbance [41]. To disentangle the complex and potentially nonlinear influences of biotic and abiotic factors on community stability, boosted regression tree (BRT), a powerful machine learning approach, is utilized [42,43]. This technique models interactions and thresholds among variables such as plant density, soil bulk density, and moisture content, allowing us to quantify the relative importance and detect the critical tipping points of environmental drivers [44,45]. Together, this integrative methodology transcends the limitations of isolated indicators, advancing a process-based understanding of plant community resilience under the patchy and stratified disturbance regime imposed by plateau zokors [31,32,46]. The framework facilitates robust ecological inference and provides actionable insights for conserving alpine grasslands facing increasing environmental pressures, such as rodent-driven soil disturbance; overgrazing by livestock; and climate change impacts, including permafrost thawing and altered hydrology [11,21,47].

Given the system’s complexity and exploratory nature, we refrain from formulating rigid a priori hypotheses. Nonetheless, guided by ecological theory and field observations, we expect that stability may deviate from the unimodal pattern predicted by the Intermediate Disturbance Hypothesis (IDH). Instead, intense disturbance could promote resilience by favoring tolerant pioneer species and reducing competitive asymmetries [48]. Furthermore, the regulatory hierarchy of stability may shift from being biologically driven under low-disturbance conditions to being increasingly constrained by abiotic filters under high-disturbance conditions [30,49,50,51,52]. By integrating the species composition, co-occurrence network structure, and environmental context, this study advances our understanding of ecological stability under spatially heterogeneous disturbance regimes and offers science-based insights for the conservation and management of alpine grasslands in the face of environmental change.

## 2. Results

### 2.1. Variation in Plant Community Stability Across Disturbance Intensities

**Figure 1:**
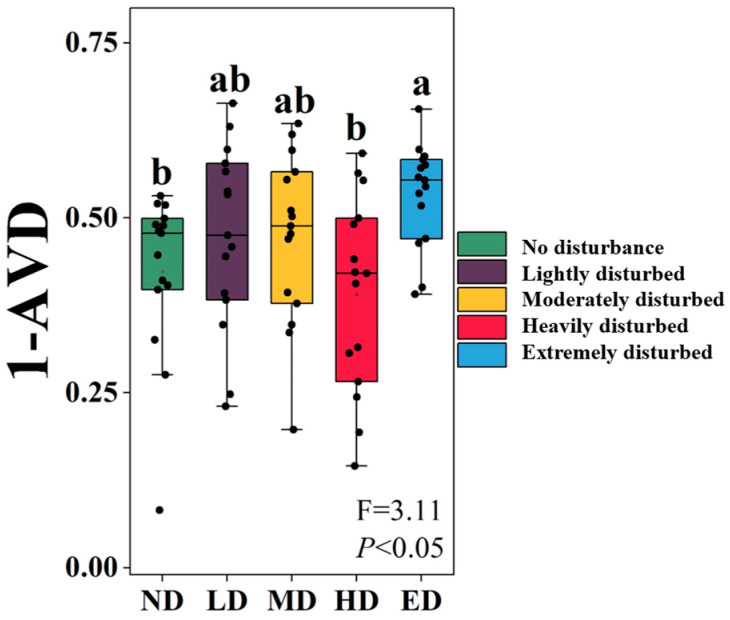
Variation in plant community stability (1−AVD) under five zokor disturbance intensities. ND: no disturbance; LD: lightly disturbed; MD: moderately disturbed; HD: heavily disturbed; ED: extremely disturbed. AVD: average variation degree. The gray horizontal line indicates the overall mean of 1−AVD across treatments. Different lowercase letters indicate statistically significant differences among disturbance levels (ANOVA, *p* < 0.05).

Plant community stability, quantified as the inverse of the average variation degree (1−AVD), varied significantly among zokor disturbance levels (Figure 1). Specifically, stability under extreme disturbance (ED) was significantly greater than that observed under no-disturbance (ND) and heavy-disturbance (HD) conditions (*p* = 0.014 and *p* = 0.002). However, comparisons among ND, lightly disturbed (LD), moderately disturbed (MD), and HD treatments did not yield sufficient evidence to reject the null hypothesis of no difference (*p* = 0.065–0.980).

### 2.2. Cohesion Indices of Plant Communities

**Figure 2:**
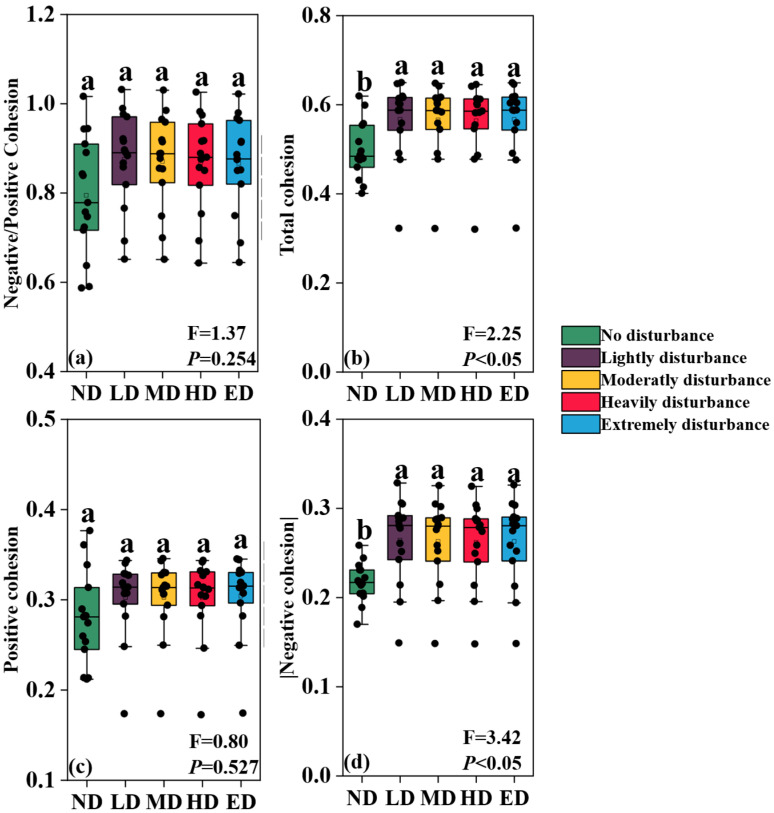
Changes in cohesion indices of plant communities across disturbance intensities. (**a**) Ratio of negative to positive cohesion; (**b**) total cohesion (sum of absolute values of positive and negative cohesion); (**c**) positive cohesion; (**d**) absolute value of negative cohesion. Different lowercase letters denote statistically significant differences among treatments (Tukey’s HSD, *p* < 0.05). Gray horizontal lines represent median values within each violin plot.

The ratio of negative to positive cohesion remained statistically consistent across all disturbance intensities (Figure 2a). Total cohesion, defined as the combined magnitude of positive and negative cohesion, exhibited a pattern closely mirroring that of negative cohesion (Figure 2b,d), largely influenced by the stability of the positive cohesion values across treatments (Figure 2c). The absolute value of negative cohesion under no disturbance (ND) was significantly lower than that observed at other disturbance levels (*p* < 0.05), whereas comparisons among the remaining treatments did not provide sufficient evidence to reject the null hypothesis of no difference.

### 2.3. Indicator Species at Different Disturbance Intensities

**Figure 3:**
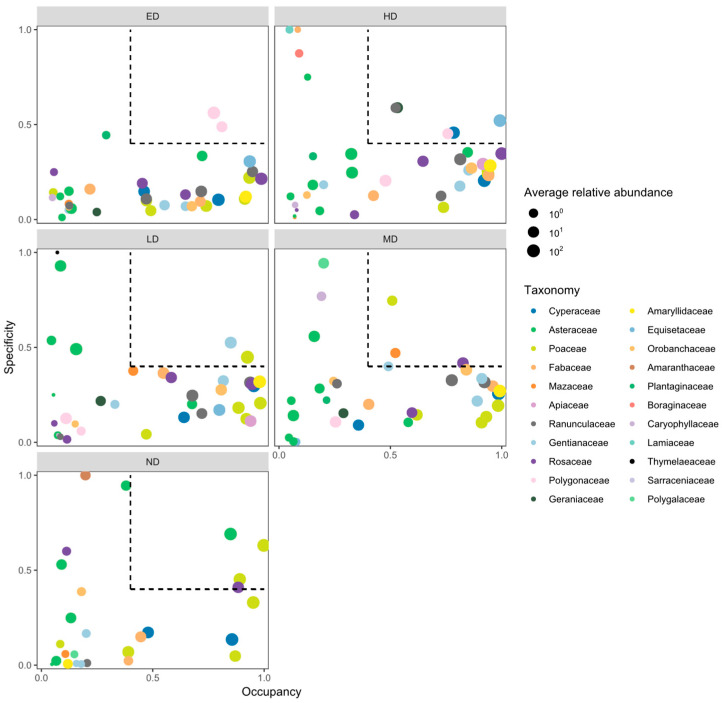
Specificity–occupancy (SPEC–OCCU) plots identifying indicator species across disturbance intensities. The *x*-axis represents occupancy, i.e., how well the plant species are distributed across all 15 quadrats of each disturbance intensity, and the *y*-axis represents specificity, i.e., whether they are also found under other disturbance intensities. Key indicator species are annotated for each disturbance level. ND, LD, MD, HD, and ED represent increasing levels of zokor disturbance.

According to the SPEC–OCCU analysis (Figure 3), the indicator species composition varied across disturbance levels (Table A1). Under no disturbance (ND), four indicator species were identified, with *Leymus secalinus* exhibiting the highest specificity and occupancy. Under light disturbance (LD), two species—including *Stipa capillata*—were recognized as indicators. Four indicator species were detected under moderate disturbance (MD), among which *Ranunculus tanguticus* and *Sibbaldianthe bifurca* demonstrated the strongest specificity. Similarly, four species were identified under heavy disturbance (HD), with *Carex atrata* being the most prominent. Under extreme disturbance (ED), two species—*Bistorta vivipara* and *Knorringia sibirica*—were identified as disturbance-specific indicators. Phytosociologically, the ND and LD plots correspond to *Kobresia humilis*-dominated alpine meadows with a high graminoid cover and relatively stable species composition. The MD plots reflect a transitional assemblage resembling *Kobresia–Stipa* meadow types, whereas the HD and ED plots are characterized by ruderal sedge–forb communities dominated by disturbance-tolerant taxa such as *Bistorta vivipara* and *Carex atrata*. These shifts align with established alpine meadow phytocoenosis classifications on the eastern Qinghai–Tibet Plateau [47,53].

### 2.4. Environmental Drivers of Plant Community Stability

**Figure 4:**
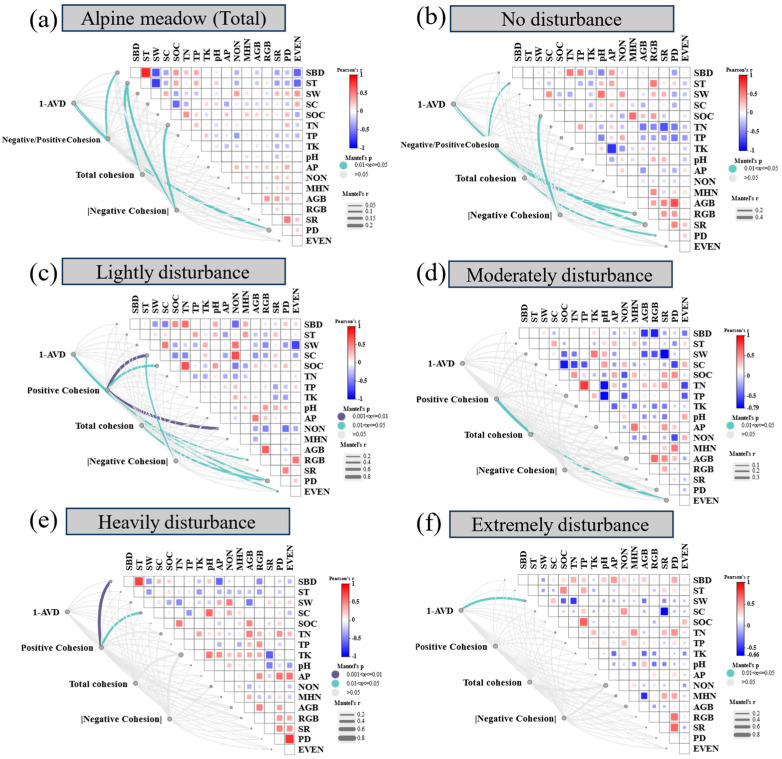
Correlation heatmaps between plant community stability metrics (1−AVD and cohesion indices) and environmental variables at each disturbance level. Variables include the following: SBD: soil bulk density; ST: soil temperature; SW: soil water content; SC: soil compaction; SOC: soil organic carbon; TN: soil total nitrogen; TP: soil total phosphorus; TK: soil total potassium; pH: soil pH; AP: soil available phosphorus; NON: soil nitrate nitrogen; MHN: soil ammonium nitrogen; AGB: aboveground biomass; RGB: belowground biomass; SR: species richness; PD: plant density; EVEN: Camargo evenness index. Significant correlations (*p* < 0.05) are highlighted.

**Figure 5:**
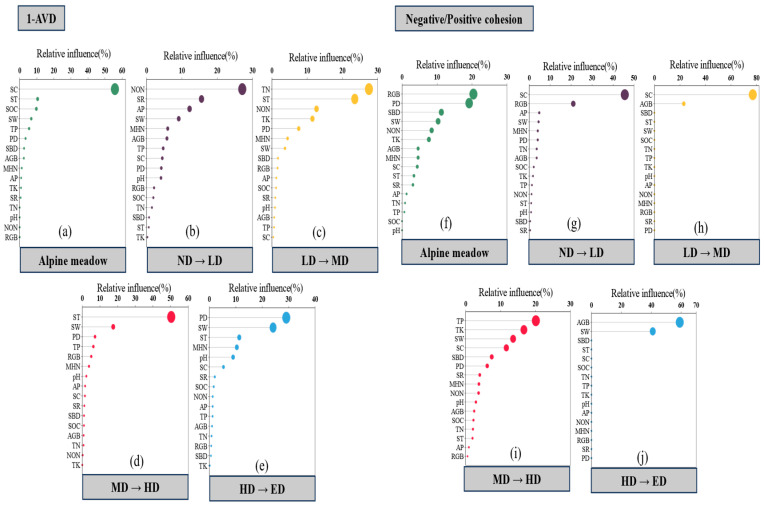
The key factors regulating plant community stability (1-AVD: (**a**–**e**); community cohesion index: (**f**–**j**)) between two disturbance intensities in a gradient of disturbance intensity. The analysis reveals shifts in dominant biotic and abiotic drivers along the disturbance gradient.

Plant community stability and cohesion metrics were significantly influenced by both biotic and abiotic factors, with their relative contributions varying across the disturbance gradient (Figure 4 and Figure 5). Across all treatments, plant density (PD) showed a consistent positive association with the stability index (1−AVD), indicating the importance of vegetation structure in maintaining compositional stability. In contrast, the ratio of negative to positive cohesion was primarily influenced by soil bulk density (SBD), while total cohesion—representing the overall strength of species co-occurrence networks—was mainly driven by total nitrogen (TN) and SBD (Figure 4a). These patterns suggest that both biological attributes and soil physical–chemical properties jointly regulate community-level stability.

Under specific disturbance levels, the identity and influence of dominant drivers shifted substantially. Under no disturbance (ND), community stability was closely associated with PD, species richness (SR), and belowground biomass (RGB), while cohesion metrics were primarily linked to soil temperature (ST) and soil organic carbon (SOC) (Figure 4b). Lightly disturbed (LD) conditions showed similar patterns, with RGB remaining a key driver of 1−AVD and cohesion being influenced by PD, SOC, and soil compaction (SC) (Figure 4c). In moderately disturbed (MD) plots, species evenness (EVEN) was the only variable significantly correlated with cohesion (Figure 4d), highlighting a shift in the role of community structure. Under heavy (HD) and extreme disturbance (ED), both stability and cohesion were predominantly governed by abiotic constraints—particularly SBD, SC, the soil water content (SW), and aboveground biomass (AGB)—indicating a transition from biotic to abiotic control as disturbance intensifies (Figure 4e,f).

Transitions between adjacent disturbance levels further revealed a dynamic reordering of environmental drivers (Figure 5). From ND to LD, increases in 1−AVD were linked to nitrate nitrogen (NON), SR, and available phosphorus (AP), while cohesion variation was associated with SC and RGB (Figure 5b,g). The shift from LD to MD saw 1−AVD responding primarily to TN, ST, NON, and total potassium (TK), with cohesion influenced by SC and AGB (Figure 5c,h). In the MD–HD transition, 1−AVD was strongly affected by ST and SW, while cohesion remained driven by AGB and SW (Figure 5d,i). From HD to ED, PD and SW became the dominant drivers of stability, while cohesion was again closely tied to AGB and SW (Figure 5e,j). These results underscore the shifting balance between biotic and abiotic regulation along the disturbance continuum and suggest that abiotic filtering becomes increasingly decisive under high-intensity zokor disturbance.

## 3. Discussion

### 3.1. Extreme Zokor Disturbance Enhances Plant Community Stability via Compositional Convergence

Contrary to long-standing ecological expectations, our findings reveal that plant community stability—measured by the inverse of the average variation degree (1–AVD)—peaks under conditions of extreme zokor disturbance (Figure 1). This result challenges the predictions of the Intermediate Disturbance Hypothesis (IDH), which posits that maximum biodiversity and community stability arise at intermediate levels of disturbance due to a trade-off between competitive exclusion and disturbance-induced mortality [12,15,54,55]. However, the IDH is grounded in assumptions of spatially uniform or stochastic disturbance regimes, whereas zokor-induced disturbance is inherently patchy, recurrent, and spatially heterogeneous, resulting in a markedly different disturbance dynamic [12,56].

In our system, extreme disturbance appears to function as a strong deterministic filter, promoting structural convergence in community composition. Under repeated soil inversion and surface disruption, only a limited subset of stress-tolerant species—characterized by clonal propagation, reduced reliance on seedling recruitment, and high belowground biomass allocation—can persist [57,58,59,60]. This leads to compositional homogenization and reduced species turnover, thereby enhancing temporal stability. Importantly, this stability is not driven by high species diversity or compensatory dynamics but rather by a form of “assembly closure,” in which persistent abiotic filtering constrains community composition within narrow ecological bounds [50,61].

The observed increase in the absolute value of negative cohesion under all disturbed conditions, relative to in undisturbed plots (Figure 2d), further supports the notion that disturbance intensifies the deterministic structuring of species interactions. Negative cohesion, often indicative of strong competitive or exclusive interactions, suggests a decline in stochastic assembly processes and an increase in structured co-occurrence under harsh environmental filters [62,63,64]. This reinforces a form of simplified but stable community organization. Such outcomes are consistent with observations in other disturbance-adapted systems, including early-successional volcanic substrates and heavily grazed grasslands [65,66,67], and they align more closely with alternative stability paradigms. These frameworks emphasize compositional rigidity and resistance to change—rather than dynamic balance—as primary mechanisms of ecological stability under sustained environmental stress [6,23,24,68].

### 3.2. Indicator Species Reflect Disturbance-Induced Regime Shifts in Community Identity

The turnover of indicator species along the zokor disturbance gradient (Figure 3; Table A1) reveals a sequence of discrete shifts in community identity rather than a gradual compositional drift. In undisturbed plots (ND), the vegetation is dominated by competitive perennial grasses such as Leymus secalinus. In contrast, communities subjected to extreme disturbance (ED) are characterized by the prevalence of pioneer or stress-tolerant species, notably Bistorta vivipara and Knorringia sibirica. Moreover, the number of indicator species declines substantially under ED conditions, indicating a convergence toward functionally redundant assemblages shaped by strong environmental filtering [19,27,57].

This abrupt transition in species identity and functional composition suggests the existence of alternative stability regimes separated by ecological thresholds—a phenomenon increasingly documented in spatially heterogeneous systems governed by feedback-driven reorganization [69,70,71]. The emergence of only two high-specificity indicator species under ED further implies that these communities may have crossed a compositional tipping point, beyond which opportunities for recolonization or stochastic reassembly become highly constrained [72]. This interpretation is corroborated by the reductions in both species richness and evenness observed in high-disturbance plots (Table A1), reflecting a collapse in functional response diversity [73].

Importantly, these indicator species may also serve as valuable tools for ecological monitoring. Their presence can act as early warning signals for disturbance-induced regime shifts, often preceding observable changes in total biomass or even diversity [74]. For instance, the increasing dominance of *Carex atrata* or *Bistorta vivipara* may indicate that a plant community has entered a degraded yet compositionally stable state—one that is unlikely to recover through passive succession alone and may require targeted soil amelioration or disturbance reduction rather than species-focused interventions [40,70]. From a phytocoenological perspective, these disturbance-induced shifts represent transitions from competitive, late-successional *Kobresia–Leymus* meadow formations toward early-successional, disturbance-adapted sedge–forb assemblages. Such transitions are consistent with the vegetation classification frameworks applied across the Qinghai–Tibet Plateau and highlight the role of abiotic filtering in driving structural convergence in degraded alpine meadows.

### 3.3. Disturbance Intensity Mediates a Shift in the Drivers of Community Stability

Our analyses reveal a marked shift in the relative importance of biotic and abiotic factors in regulating plant community stability across the zokor disturbance gradient (Figure 4). Under low-intensity disturbance conditions (ND–LD), community stability—as indicated by the inverse of the average variation degree (1–AVD)—is most strongly associated with plant density (PD), species richness (SR), and belowground biomass (RGB). These findings suggest that vegetation structure confers stability through internal buffering mechanisms such as spatial complementarity, biomass allocation, and functional redundancy [27,75].

However, as disturbance intensity increased, the predictive power of biotic variables weakened, while soil physical properties—particularly soil bulk density (SBD), compaction (SC), and moisture content (SW)—emerged as the dominant drivers of both compositional stability and species co-occurrence cohesion (Figure 4e,f). This shift from biotic to abiotic control was further substantiated by results from boosted regression tree (BRT) models comparing adjacent disturbance levels (Figure 5). Plant-based predictors such as RGB and PD dominate under transitions from ND to MD, whereas abiotic predictors increasingly govern community dynamics during transitions from HD to ED [42]. Notably, species richness exhibited a strong positive association with stability under no- and light-disturbance conditions (ND–LD), suggesting that higher richness enhanced stability via mechanisms such as functional redundancy and spatial complementarity. However, this relationship diminished progressively with an increasing disturbance intensity, becoming negligible or even slightly negative under heavy- and extreme-disturbance (HD–ED) levels. In these latter stages, stability was achieved despite reduced richness, primarily through deterministic filtering and dominance of disturbance-tolerant taxa. This pattern indicates a shift from diversity-dependent stability to diversity-independent stability along the disturbance gradient.

These patterns suggest the existence of control regime thresholds, wherein the stabilizing influence of vegetation structure is progressively overridden by physical habitat constraints [76,77]. Such threshold effects are consistent with trait-based frameworks of ecosystem assembly and stability, which propose that biotic regulation gives way to abiotic filtering as environmental stress increases [27,78]. In applied terms, this implies that, beyond a certain disturbance intensity, biodiversity alone may be insufficient to ensure ecosystem resilience, and active remediation of soil conditions may be necessary to restore functional stability [70,78].

Our findings also align with emerging ecological resilience theory, which distinguishes between resistance—the capacity to withstand change—and resilience—the capacity to recover following disturbance [79]. While communities under extreme disturbance may exhibit apparent compositional stability, they may be locked into low-resilience regimes dominated by structural rigidity and diminished response diversity [73,80]. In such systems, the loss of functional flexibility may render them vulnerable to further degradation or collapse under additional stressors.

### 3.4. Practical Implications for Alpine Grassland Management Under Zokor Disturbance

Our findings provide field-based evidence that disturbance intensity fundamentally alters the ecological pathways through which plant community stability is maintained in alpine ecosystems. In contrast to classical predictions that stability peaks at intermediate disturbance levels, our results show that the highest compositional stability occurred under extreme zokor disturbance (Figure 1). This pattern suggests that structurally simplified, disturbance-filtered communities can achieve stability through resistance to further change, even in degraded environmental contexts [69,81]. Such findings challenge the conventional assumption of a positive correlation between disturbance intensity and ecological instability [54,55].

From a management perspective, the observed shift in dominant stability drivers—from plant-based traits such as density and belowground biomass at low disturbance to soil physical properties under severe disturbance (Figure 4)—implies the need for disturbance stage-specific interventions [82,83]. In the early phases of disturbance, strategies that enhance plant structural complexity (e.g., promoting species richness and increasing root biomass) may strengthen internal buffering mechanisms and improve resistance to further disruption [7,27]. In contrast, under extreme disturbance, biological recovery is likely constrained by abiotic degradation, necessitating soil-targeted restoration approaches such as decompaction, hydrological improvement, or organic amendment to facilitate recolonization and functional recovery [77,84].

Additionally, the identification of disturbance-specific indicator species (Figure 3; Table A1) offers a valuable bio-diagnostic tool for ecosystem monitoring. Species such as *Bistorta vivipara* and *Knorringia sibirica*, which dominate under extreme disturbance, may signify that a community has crossed a threshold into a low-diversity, rigidity-dominated regime. Their persistence can signal limited regenerative potential and serve as early warning indicators of regime lock-in, particularly when incorporated into long-term monitoring frameworks [74,85]. Such indicators are crucial for distinguishing between transient states and stabilized configurations, as well as for informing whether passive recovery remains viable or whether active intervention is warranted [70,72].

Together, these insights do not demand a wholesale revision of disturbance–stability theory, but they do call for its refinement in the context of spatially heterogeneous, engineer-driven systems such as those shaped by zokor activity. Our results emphasize that stability can arise from compositional convergence rather than diversity per se and that such stability—though persistent—may be ecologically undesirable if it reflects low resilience or functional stagnation [25,78,86]. Effective management of alpine meadows under zokor disturbance therefore requires more than population control; it demands recognition of functional thresholds, ecological legacies, and the context-dependent nature of stability itself.

### 3.5. Limitations and Future Perspectives

Although this study advances the understanding of how plateau zokor disturbance influences alpine plant community stability, certain limitations should be acknowledged. First, the disturbance gradient was inferred from mound coverage and spatial configuration at a single time point, without accounting for interannual variations in disturbance intensity or historical disturbance legacies. Temporal fluctuations in disturbance regimes can strongly influence community trajectories and stability metrics, as shown in long-term alpine monitoring studies [87,88]. Second, our analyses focused on taxonomic composition and species co-occurrence networks; incorporating functional traits and phylogenetic diversity [89,90] would provide a more mechanistic understanding of how disturbance filters species and functions. Similarly, belowground biotic interactions, such as soil microbial communities and mycorrhizal networks, play critical roles in mediating recovery and stability under disturbance [30] yet were not assessed here. Third, the observational design limits causal inference between abiotic constraints and stability outcomes. Experimental manipulations, such as controlled soil compaction or hydrological adjustments, have been shown to clarify cause–effect relationships in alpine systems [91,92].

Future research should therefore (i) integrate multi-year and multi-season data to capture temporal variability [93]; (ii) expand spatial coverage to include multiple alpine meadow types across the Tibetan Plateau [47]; (iii) incorporate trait-based and belowground biodiversity metrics; and (iv) combine observational and experimental approaches to disentangle the relative contributions of biotic and abiotic drivers. Such efforts will refine disturbance–stability theory in patchy alpine systems and inform more targeted management strategies under accelerating environmental change.

## 4. Materials and Methods

### 4.1. Sites Description

**Figure 6:**
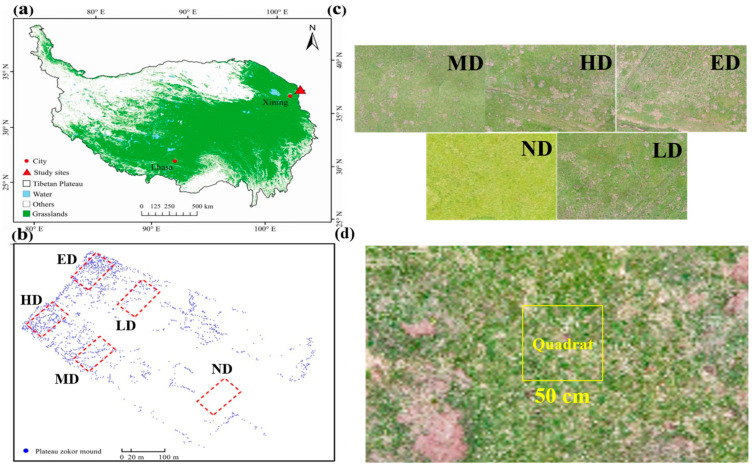
Map of the studied regions and illustration of experimental design. (**a**): Locations of the study site; (**b**): Spatial distribution map of plateau zokor mounds; (**c**): Aerial views of landscape distributed by zokor mound in five different disturbance levels. (**d**): sample plot setting and sampling site distribution.

This study was conducted in an alpine meadow ecosystem located on the northeastern margin of the Qinghai–Tibet Plateau (37°12′ N, 102°46′ E; elevation: 2937 m), within Tianzhu County, Gansu Province, China (Figure 6a). The region is characterized by a typical alpine continental climate, with a mean annual temperature of –0.1 °C, mean annual precipitation of 416 mm, and average annual evaporation of 1592 mm. The dominant soil type is alpine chernozem, featuring a surface root mat approximately 5–10 cm thick. This dense mat, primarily formed by the intertwined root systems of sedges and grasses, acts as an insulating barrier that reduces water infiltration and buffers soil temperature fluctuations, thereby playing a critical role in mitigating permafrost thawing [47].

The vegetation is dominated by *Kobresia pygmaea*, accompanied by subordinate species, including *Elymus nutans*, *Kobresia capillifolia*, *Poa crymophila*, and *Carex atrofusca*. The plateau zokor (*Eospalax baileyi*) is the only mound-building subterranean rodent that has been recorded in this area over the past two decades [14]. Through repeated burrowing and soil excavation, zokors generate a complex mosaic of mounds and inter-mound areas that fundamentally reshape the meadow’s microtopography. These physical alterations cause a significant redistribution of soil moisture, temperature, and nutrient conditions, which, in turn, drive pronounced shifts in plant community composition and structure across the landscape.

### 4.2. Experimental Design

Previous studies have commonly quantified the disturbance intensity of burrowing rodents by analyzing the spatial configuration of mound patches, thereby assessing the ecological impacts of their burrowing and mound-building activities [94,95,96]. Following this approach, we classified zokor disturbance into five intensity levels based on the proportion and spatial distribution of zokor mounds within the landscape (Figure 6b) [96]: (1) no disturbance (ND)—grassland with no visible zokor mounds; (2) light disturbance (LD)—a mound coverage of 16.25%; (3) moderate disturbance (MD)—a mound coverage of 23.61%; (4) heavy disturbance (HD)—a mound coverage of 29.25%; and (5) extreme disturbance (ED)—a mound coverage reaching 35.70% (Figure 6c; Table 1). Each disturbance level was represented by three replicate plots (20 m × 20 m each), with a minimum spacing of 100 m between plots of different disturbance levels to ensure spatial independence and avoid edge effects. Within each plot, five fixed sampling points were established and permanently marked using numbered wooden stakes. These sampling points were used for vegetation relevés and to collect plant and soil samples for physicochemical analysis.

### 4.3. Field Relevé and Sampling

To assess plant community characteristics across different levels of zokor disturbance, a soil monolith measuring 0.5 m × 0.5 m × 0.2 m was excavated at each permanently marked sampling point (Figure 6d). All plant individuals within the sampling frame were carefully collected by hand and identified at the species level in the field or laboratory. Species were identified in the field with reference to the Chinese Virtual Herbarium (Institute of Botany, the Chinese Academy of Sciences, Beijing) and subsequently verified by taxonomic specialists to ensure the accuracy and reliability of their determination. Species richness (SR) was recorded as the total number of species per quadrat, and species abundance was documented as the number of individuals per species. After identification and counting, plant materials were separated into aboveground and belowground components. Roots were carefully washed with clean water to remove adhered soil particles. All samples were stored in pre-labeled paper envelopes and oven-dried at 75 °C for 48 h to determine aboveground biomass (AGB) and belowground biomass (BGB).

### 4.4. Soil Physicochemical Analysis

Following vegetation relevés, soil measurements and sampling were conducted at the same fixed sampling points corresponding to each disturbance level. A total of twelve soil environmental variables were assessed, comprising four physical and eight chemical parameters. Soil temperature and moisture at a depth of 5–20 cm were measured in situ using TZS-IIW soil temperature and moisture sensors (Top Instrument Co., Zhejiang, China). Soil compaction (SC) was quantified with an SC-900 soil compaction meter (Spectrum Technologies, Aurora, IL, USA). Soil pH was determined using a digital pH meter (ST2100, OHAUS, Shanghai, China). Chemical properties were analyzed following standardized protocols [98]. Soil organic carbon (SOC) was measured via the dichromate oxidation method using K_2_Cr_2_O_7_–H_2_SO_4_ [98]. Total nitrogen (TN) was quantified using the Kjeldahl digestion method [98]. Total phosphorus (TP) and available phosphorus (SP) were measured using the molybdenum–antimony colorimetric method [98]. Total potassium (TK) was determined with a flame photometer (FP6431, YD Ltd., Shanghai, China) following dry ashing. Nitrate nitrogen (NON) and ammonium nitrogen (NHN) concentrations were extracted with 2 mol L^−1^ KCl and analyzed using an automated flow injection analyzer (FS3100, O.I. Corporation/Xylem Inc., College Station, TX, USA).

### 4.5. Community Indicator Calculations

#### 4.5.1. Average Variation Degree (AVD)

Plant community stability was evaluated using the average variation degree (AVD) [33,99], which quantifies the deviation of plant species abundance from the mean across different disturbance intensities. A lower AVD value indicates a higher level of community stability. The variation degree for each species was calculated using the following equation:

(1) $$\left|a_{i}\right|=\frac{\left|x_{i}-{\bar{x}}_{i}\right|}{\delta_{i}}$$

where $a_{i}$ represents the variation degree of species *i*; $x_{i}$ is the abundance of species *i* in a single sample under a specific disturbance intensity; ${\bar{x}}_{i}$ is the mean abundance of species *i* calculated across all samples under the same disturbance level; and $\delta_{i}$ is the standard deviation of species *i*’s abundance under that disturbance condition. Since $x_{i}$, ${\bar{x}}_{i}$, and $\delta_{i}$ share the same unit (individual count), the resulting $a_{i}$ is a dimensionless, standardized measure of how far the species abundance in a given sample deviates from the mean relative to the overall variability. Lower values of $a_{i}$ reflect greater stability in species abundance.

The AVD values were calculated using the following equation:

(2) $$AVD=\frac{\sum_{i=1}^{n}\left|a_{i}\right|}{K\times n}$$

where *k* is the number of samples in one disturbance condition, and *n* is the number of species in each disturbance condition.

#### 4.5.2. Community Cohesion

Community cohesion was quantified to evaluate the overall strength of species associations within plant communities. The cohesion index reflects the extent of positive or negative interactions among species based on statistically significant pairwise correlations in abundance data. This approach captures potential biotic interactions, such as facilitation or competition, which may contribute to community stability [63,100,101]. Cohesion was calculated separately for positive and negative interactions using the following equation:

(3) $${Conhension}_{j}^{\pm }=\sum_{i=1}^{n}a_{ij}\cdot {{\bar{r}}_{i}}^{\pm }$$

(4) $$Network\;stability=\frac{\left|Negaitive\;cohesion\right|}{Positive\;cohesion}$$

where ${Conhension}_{j}^{+}$ and ${Conhension}_{j}^{-}$ represent the positive and negative cohesion scores for sample *j*, respectively. $a_{ij}$ is the abundance of species “i” in sample “j”. ${{\bar{r}}_{i}}^{\pm }$ is the average of all significant positive (or negative) Pearson correlation coefficients between species *i* and all other species across the dataset. The values of positive cohesion range from 0 to 1, while the negative cohesion values fall between –1 and 0. High positive cohesion indicates strong co-occurrence (e.g., facilitation), whereas high negative cohesion reflects strong segregation or competition among species. Notably, communities exhibiting greater negative cohesion are generally considered more stable, as they tend to maintain lower β-diversity across environmental, spatial, or temporal gradients, reflecting compositional consistency under variable conditions.

#### 4.5.3. Specificity and Occupancy

To identify species indicative of particular disturbance intensities, we applied the specificity and occupancy (SPEC–OCCU) framework proposed by Dufrêne and Legendre [39]. This method quantifies a species’ ecological fidelity to a specific habitat or treatment level based on two complementary metrics: specificity and occupancy [102]. Specificity reflects the concentration of a species’ abundance at a given disturbance level and is calculated as the mean abundance of species *S* in habitat *H*, divided by its mean abundance across all habitats. A higher value indicates that the species occurs predominantly, or almost exclusively, in that specific habitat. Occupancy represents the consistency of species occurrence and is defined as the proportion of plots within *H* where species *S* is present. It captures the spatial fidelity of species distribution across sampling units. Together, these two metrics enable the identification of high-fidelity indicator species that are both frequent and abundant within a specific disturbance context, thus serving as ecological signals of regime shifts or community transitions.

(5) $$Specificity=\frac{N{individuals}_{S,H}}{N{individuals}_{S}}$$

(6) $$Occupancy=\frac{N{sites}_{S,H}}{N{sites}_{H}}$$

The specificity of plant species *S* in disturbance *H* is calculated as the ratio of the average abundance of *S* in all quadrats of *H* ($N{individuals}_{S,H}$) to the sum of the average abundance of *S* at all disturbance intensities in this study ($N{individuals}_{S}$). *H* is calculated as the ratio of the number of *S* samples ($N{sites}_{S,H}$) to the total number of *H* quadrats ($N{sites}_{H}$). Specificity–occupancy plots were generated for visualization purposes. In this study, to identify species that were specific to each disturbance intensity, we selected plant species with specificity and occupancy values greater than or equal to 0.4 based on the method followed by Gweon et al. [102].

### 4.6. Statistical Analysis

All statistical analyses were conducted using SPSS version 22.0 (IBM, USA) [103] and R version 4.2.3 (R Core Team, Vienna, Austria) [104]. First, a one-way analysis of variance (ANOVA) was used to test the effects of different zokor disturbance intensities on plant community stability (measured using the average variation degree, AVD) and cohesion metrics, including the ratio of negative to positive cohesion, total cohesion, positive cohesion, and the absolute value of negative cohesion. Statistical significance was determined at *p* < 0.05. Second, Pearson correlation analyses were performed using the “ggcor” package to explore the relationships between the AVD, cohesion indices, and biotic and abiotic variables at each disturbance level. Significance was again assessed at *p* < 0.05. Third, to identify the key drivers of the AVD and cohesion across and within disturbance levels, we employed boosted regression tree (BRT) modeling using the “gbm” package. This method enables the detection of nonlinear and interactive effects among predictor variables. Data visualization was carried out using Origin 2023b (OriginLab Corporation, Northampton, MA, USA), custom Python scripts, and the “*plotnine*” package [105].

## 5. Conclusions

Our study reveals that, in zokor-disturbed alpine grasslands, plant community stability peaks under extreme rather than intermediate disturbance—challenging classical disturbance–stability models. This stability arises not from enhanced diversity but from deterministic filtering and compositional convergence, resulting in structurally simplified yet persistent communities. We show that the drivers of stability shift along the disturbance gradient: biotic factors (e.g., plant density and belowground biomass) dominate under low disturbance, while abiotic constraints (e.g., soil compaction and moisture) become critical under high disturbance. These findings highlight the need for stage-specific management—enhancing plant structure at early stages and focusing on soil restoration at later ones. Moreover, disturbance-specific indicator species offer early diagnostic signals of regime shifts, with implications for monitoring and intervention. Overall, our results underscore that stability does not always indicate resilience and that persistent states under extreme disturbance may reflect ecological rigidity. In such rigid systems, high vulnerability and pronounced spatiotemporal variations can amplify sensitivity to further environmental change, accelerating species turnover and functional loss. These processes reduce the range of tolerable environmental conditions, ultimately driving rapid declines and significant contractions in suitable habitat areas. Effective conservation of alpine grasslands requires integrating disturbance thresholds, species indicators, and tailored restoration strategies.

## Figures and Tables

**Table 1:** Spatial pattern indices of plateau zokor mound patches across different disturbance levels. Values are presented as means ± SE. Different lowercase letters within the same row indicate statistically significant differences at the *p* < 0.05 level. Index calculations follow McGarigal [97].

Index	Lightly Disturbed	Moderately Disturbed	Heavily Disturbed	Extremely Disturbed
Total area of patch/m^2^	64.82 ± 1.894 c	94.46 ± 4.441 bc	117.3 ± 3.765 ab	142.81 ± 2.578 a
Total edge of patch/m	174.37 ± 6.451 c	248.01 ± 4.443 b	297.15 ± 7.546 b	411.38 ± 6.591 a
Splitting index	1.46 ± 0.016 c	1.66 ± 0.038 bc	1.94 ± 0.042 ab	2.18 ± 0.038 a
Shape index	1.13 ± 0.008 a	1.15 ± 0.002 a	1.16 ± 0.001 a	1.17 ± 0.010 a

## Data Availability

The original contributions presented in this study are included in the article. Further inquiries can be directed to the corresponding authors.

## Appendix A

**Table A1:** The indicator species in each disturbance intensity.

Disturbance Intensity	Indicator Species	Abundance_Mean
No disturbance	*Leymus secalinus*	116.87
	*Poa araratica*	55.20
	*Sibbaldianthe bifurca*	13.93
	*Artemisia frigida*	77.20
Light disturbance	*Stipa capillata*	148.53
	*Swertia tetraptera*	23.67
Moderate disturbance	*Cleistogenes squarrosa*	3.13
	*Lancea tibetica*	2.67
	*Ranunculus tanguticus*	16.80
	*Sibbaldianthe bifurca*	14.20
Heavy disturbance	*Carex atrata*	48.00
	*Knorringia sibirica*	6.60
	*Geranium pylzowianum*	7.93
	*Gymnaconitum gymnandrum*	2.67
Extreme disturbance	*Bistorta vivipara*	68.33
	*Knorringia sibirica*	7.13
